# Immunotherapy With Anti‐PD‐1 Versus Anti‐PD‐L1 in SCLC: A Comprehensive Retrospective Analysis

**DOI:** 10.1002/mco2.70872

**Published:** 2026-09-11

**Authors:** Ruiyuan Yang, Jiadi Gan, Lan Yang, Bojiang Chen, Weimin Li

**Affiliations:** ^1^ Department of Pulmonary and Critical Care Medicine West China Hospital, Sichuan University Chengdu Sichuan China; ^2^ State Key Laboratory of Respiratory Health and Multimorbidity West China Hospital Chengdu Sichuan China; ^3^ Institute of Respiratory Health, Frontiers Science Center for Disease‐Related Molecular Network West China Hospital, Sichuan University Chengdu Sichuan China; ^4^ Precision Medicine Center, Precision Medicine Key Laboratory of Sichuan Province West China Hospital, Sichuan University Chengdu Sichuan China; ^5^ The Research Units of West China Chinese Academy of Medical Sciences, West China Hospital Chengdu Sichuan China; ^6^ Institute of Respiratory Health and Multimorbidity West China Hospital, Sichuan University Chengdu Sichuan China

**Keywords:** anti‐PD‐1, anti‐PD‐L1, small cell lung cancer, survival

## Abstract

Small cell lung cancer (SCLC) is a highly malignant tumor characterized by strong invasiveness and poor prognosis. Immune checkpoint inhibitors have provided new therapeutic options for SCLC, but their comparative clinical benefit remains unclear. This retrospective study enrolled 171 patients with SCLC treated between January 2016 and June 2023, with follow‐up through June 30, 2024, to compare survival and safety profiles between anti‐programmed death‐1 (anti‐PD‐1) and anti‐programmed death‐ligand 1 (anti‐PD‐L1) inhibitors. Among the 119 patients (69.6%) who received immunotherapy plus chemoradiotherapy, the median overall survival (OS) was 48.3 months, compared with 25.2 months in those who did not receive radiotherapy. Multivariable Cox regression confirmed that liver metastasis and radiotherapy status were independently associated with OS and progression‐free survival (PFS). Subgroup analyses revealed that patients younger than 65 years who underwent radiotherapy, particularly those without liver metastasis, achieved longer OS with anti‐PD‐1 agents. In conclusion, both anti‐PD‐1 and anti‐PD‐L1 inhibitors improved the prognosis of SCLC. Patients younger than 65 years receiving radiotherapy and those with limited‐stage SCLC may represent populations more likely to benefit from anti‐PD‐1 therapy.

## Graphical abstract

1. Both PD‐1 and PD‐L1 inhibitors significantly improved overall survival (OS) and progression‐free survival (PFS). 2. Younger patients (aged < 65 years) who received radiotherapy or those with limited‐stage SCLC may be the preferred population for anti‐PD‐1 treatment in SCLC

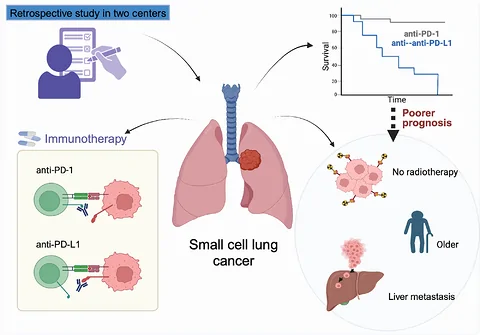

## 1 Introduction

Small cell lung cancer (SCLC) is a highly aggressive subtype of lung cancer, accounting for approximately 15% of all lung cancer cases globally and remaining a leading cause of cancer‐related death [1]. It is characterized by high metastatic potential and early treatment resistance. Despite its initial sensitivity to chemotherapy (CT) and radiotherapy (RT), the 5‐year survival rate remains less than 10% because of frequent recurrence and rapid progression [2]. SCLC is clinically classified into limited‐stage SCLC (LD‐SCLC) and extensive‐stage SCLC (ED‐SCLC) based on tumor extension. Tobacco smoking is the strongest and most well‐established epidemiological risk factor, accounting for more than 90% of cases. Among nonsmokers, air pollutants such as PM10 and NO_2_ have been implicated in elevated lung cancer risk, although clinical evidence remains limited [3, 4].

For decades, platinum‐based CT combined with thoracic RT has been the mainstay of SCLC treatment [5]. Although initial tumor response rates can range from 60% to 80%, most patients experience relapses within 6 to 12 months, creating an urgent need for more effective therapeutic approaches. In recent years, immune checkpoint inhibitors (ICIs) have emerged as transformative treatments and have significantly extended survival in patients with SCLC [6, 7].

Numerous clinical trials have verified the clinical value of anti‐programmed death‐1 (anti‐PD‐1) and anti‐programmed death‐ligand 1 (anti‐PD‐L1) agents in SCLC, thereby reshaping the treatment pattern for this aggressive disease. For ED‐SCLC, pivotal studies such as IMpower133 and CASPIAN established anti‐PD‐L1 therapy combined with CT as a preferred first‐line standard of care. In addition, ASTRUM‐005 demonstrated that anti‐PD‐1 therapy provides survival benefit in Asian patients with ED‐SCLC when used as first‐line therapy, helping address the need for region‐specific treatment evidence [8].

For second‐line and subsequent treatment, nivolumab and pembrolizumab, both anti‐PD‐1 agents, have been frequently used in clinical practice. Nivolumab monotherapy has been evaluated for metastatic SCLC after two or more prior lines of therapy, and Ready et al. reported that nivolumab achieved a higher objective response rate, particularly when combined with ipilimumab [9]. In KEYNOTE‐028 and KEYNOTE‐158, pembrolizumab showed promising clinical benefit in patients with previously treated recurrent or metastatic SCLC, supporting its use as a subsequent treatment option irrespective of PD‐L1 expression levels [10, 11].

Notably, most prior studies have compared anti‐PD‐1 or anti‐PD‐L1 therapy with CT or placebo, whereas direct comparisons between these two ICI classes remain scarce. This evidence gap restricts clinicians' ability to make optimal individualized treatment decisions, especially regarding which ICI type may be more effective or better tolerated in specific subgroups. Therefore, this two‐center retrospective study analyzed 171 patients with SCLC to compare survival outcomes, safety profiles, and subgroup responses between anti‐PD‐1 and anti‐PD‐L1 therapies. We found no significant survival difference in the overall cohort, but anti‐PD‐1 therapy appeared to provide greater OS benefit in selected populations, including younger patients receiving RT and those with LD‐SCLC, highlighting the potential importance of individualized ICI selection in SCLC.

## 2 Results

### 2.1 Clinical Characteristics

**FIGURE 1:**
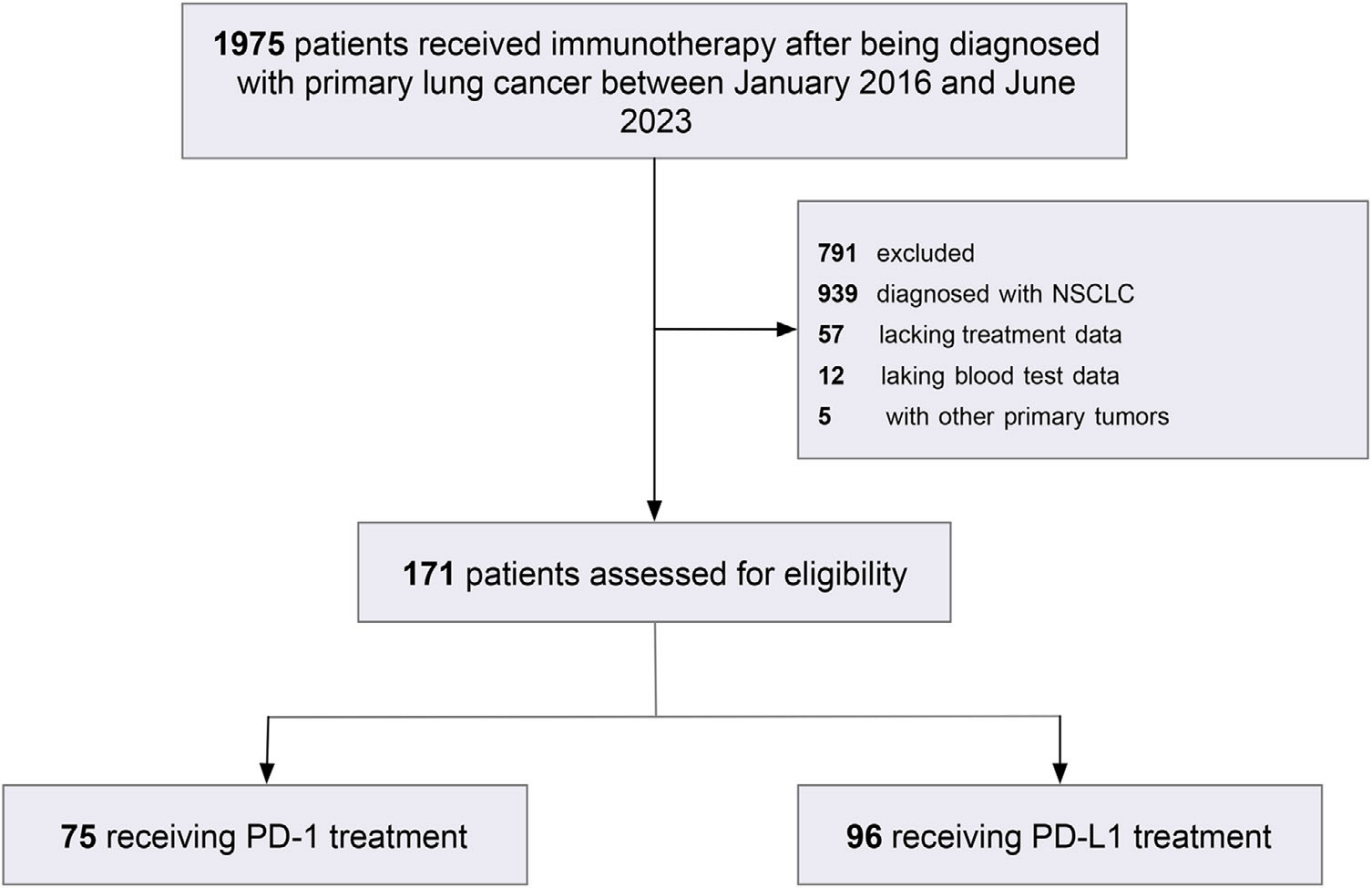
Flow diagram of patient selection. From the initial cohort, patients were screened according to the predefined inclusion and exclusion criteria, and 171 eligible patients were included in the final analysis.

We herein characterized the baseline clinical characteristics and treatment distribution of the included patients. From an initial cohort of 1975 patients, 171 were eligible for the final analysis, including 75 patients in the anti‐PD‐1 group and 96 patients in the anti‐PD‐L1 group (Figure 1). As shown in Table 1, the median age was 62.6 years in the anti‐PD‐1 group and 62.1 years in the anti‐PD‐L1 group. In the anti‐PD‐1 group, 63 patients were male (84.0%), 74.7% had a history of smoking, and 70.7% had ED‐SCLC. The most frequent metastatic sites were the lung (49.0%), liver (16.0%), and bone (16.0%). Ki‐67 expression was less than 15% in 28.0% of patients and 15% or greater in 21.3% of patients. PD‐L1 tumor proportion score (TPS) data were available for a limited subset. All patients in this group received combination therapy, with IT plus CT and RT accounting for 62.7%. The baseline clinical and demographic characteristics of the anti‐PD‐L1 group were generally comparable with those of the anti‐PD‐1 group, and no statistically significant intergroup differences were identified (all *p* > 0.05). Overall, the two groups were well‐balanced for subsequent survival and safety comparisons.

**TABLE 1:** Patient characteristics and treatments.

	PD‐1 group (*n* = 75)	PD‐L1 group (*n* = 96)	Total (*n* = 171)	
Characteristics	Patients, No. (%)	Patients, No. (%)	Patients, No. (%)	*p* value
Age, median (SD)	62.6 (8.54)	62.1 (7.90)	62.3 (8.16)	0.424
Sex, *n* (%)				0.862
Male	63 (84.0)	87 (90.6)	150 (87.7%)	
Female	12 (16.0)	9 (9.4)	21 (12.3%)	
BMI, median (SD)	22.6 (3.01)	23.3 (2.97)	23.0 (3.00)	0.244
Smoking history				
Never	19 (25.3)	21 (21.9)	40 (23.4)	0.869
Ever	56 (74.7)	75 (78.1)	131 (76.6)	
Underlying conditions (%)				
Hypertension	9 (12.0)	23 (24.0)	32 (18.7)	0.138
Diabetes mellitus	4 (5.3)	13 (13.5)	17 (9.9)	0.205
Coronary heart disease	3 (4.0)	6 (6.3)	9 (5.3)	0.808
ECOG				0.914
0	20 (26.7)	31 (32.3)	51 (29.8)	
1	49 (65.3)	54 (56.3)	103 (60.2)	
≥ 2	6 (8.0)	7 (7.3)	13 (7.6)	
NA	0 (0.0)	4 (4.2)	4 (2.3)	
Ki‐67				0.246
< 15%	21 (28.0)	30 (31.3)	51 (29.8)	
≥ 15%	16 (21.3)	45 (46.9)	61 (35.7)	
Unknown	38 (50.7)	21 (21.9)	59 (34.5)	
PD‐L1 TPS %				0.489
< 1	11 (14.7)	0 (0.0)	11 (6.4)	
1–49	11 (14.7)	4 (4.2)	15 (8.8)	
≥ 50	5 (6.7)	1 (1.0)	6 (3.5)	
Unknown	48 (64.0)	91 (94.8)	139 (81.3)	
ED‐SCLC	53 (70.7)	80 (83.3)	133 (77.8)	0.142
Metastasis				
None	9 (12.0)	9 (9.4)	18 (10.5)	0.857
Brain	5 (6.7)	13 (13.5)	18 (10.5)	0.348
Liver	12 (16.0)	26 (27.1)	38 (22.2)	0.224
Bone	12 (16.0)	27 (28.1)	39 (22.8)	0.172
Lung	47 (49.0)	49 (65.3)	96 (56.1)	0.101
Therapy arrangement				0.429
IT	0 (0.0)	1 (0.6)	1 (0.6)	
IT + CT	26 (34.7)	23 (24.0)	49 (28.7)	
IT + RT	2 (2.7)	0 (0.0)	2 (1.2)	
IT + CT + RT	47 (62.7)	72 (75.0)	119 (69.6)	
Continuous variables				
Platelet count × 10^9^/L mean (SD)	219 (84.6)	231 (85.6)	226 (85.1)	0.594
Neutrophil count × 10^9^/L mean (SD)	5.49 (2.80)	5.35 (7.10)	5.41 (5.59)	0.272
Lymphocyte count × 10^9^/L mean (SD)	1.39 (0.61)	1.65 (2.04)	1.53 (1.57)	0.730
Monocyte count × 10^9^/L mean (SD)	8.89 (41.1)	16.3 (54.6)	13.0 (49.2)	0.996
PLR (SD)	197 (135)	208 (251)	203 (206)	0.937
NLR (SD)	5.34 (5.49)	4.01 (3.06)	4.61 (4.36)	0.317
LMR (SD)	3.54 (2.84)	4.27 (5.77)	3.94 (4.69)	0.817
Serum albumin (SD)	40.1 (5.01)	49.7 (6.29)	45.4 (4.71)	0.619
LDH (SD)	289 (474)	324 (313)	308 (392)	0.283

Abbreviations: BMI, body mass index; CT, chemotherapy; ECOG, Eastern Cooperative Oncology Group; IT, immunotherapy; PD‐1, programmed cell death 1; PD‐L1, programmed death‐ligand 1; RT, radiotherapy; TPS, tumor proportion score.

### 2.2 Survival Outcomes

**FIGURE 2:**
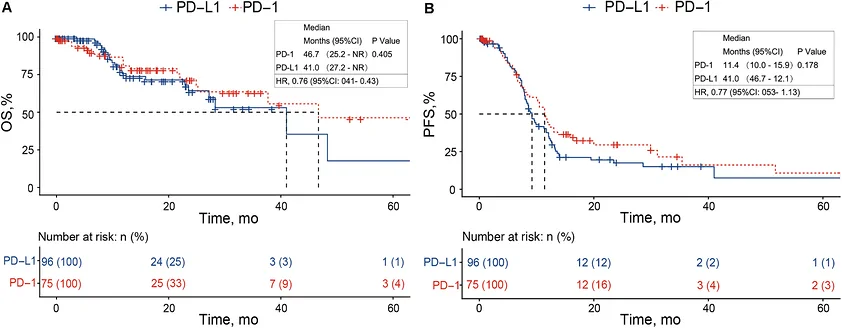
Kaplan–Meier curves comparing overall survival (OS) and progression‐free survival (PFS) between the anti‐PD‐1 and anti‐PD‐L1 groups in the entire cohort. (A) OS in patients treated with anti‐PD‑1 versus anti‐PD‑L1; (B) PFS in patients treated with anti‐PD‑1 versus anti‐PD‑L1.

Survival was compared across anti‐PD‐1 and anti‐PD‐L1 cohorts, with the prognostic effects of baseline variables and treatment modalities further evaluated. The follow‐up period ranged from 0.1 to 105.5 months. Kaplan–Meier survival curves were generated to compare OS and PFS between the two groups (Figure 2). The median OS was 46.7 months in the anti‐PD‐1 group and 41.0 months in the anti‐PD‐L1 group, and the median PFS was 11.4 months and 9.2 months, respectively. No significant between‐group differences were detected in OS (*p* = 0.405) or PFS (*p* = 0.178). Subgroup Kaplan–Meier analyses stratified by age, sex, BMI, smoking status, and underlying comorbidities are shown in Figures S1–S5. When stratified by BMI (< 25 kg/m^2^ vs. ≥ 25 kg/m^2^), anti‐PD‐L1 therapy provided a significant OS benefit in patients with BMI ≥ 25 kg/m^2^ (*p* = 0.029), although no difference was observed for PFS. Smoking status and comorbidities, including diabetes, hypertension, and coronary heart disease, did not significantly modify survival differences between the two treatment groups. Only one patient received IT monotherapy, whereas most patients (119/171, 69.6%) received IT plus CT and RT. Patients receiving triple combination therapy had a longer median OS than those not receiving RT (48.3 vs. 25.2 months; *p* = 0.007), whereas median PFS was longer in the non‐RT group (13.2 vs. 9.2 months; *p* = 0.014) (Figure S6). These findings suggest that overall survival was comparable between anti‐PD‐1 and anti‐PD‐L1 therapies in the entire cohort, while RT‐containing regimens were associated with improved OS.

### 2.3 Prognostic Analysis and Subgroup Analysis

**FIGURE 3:**
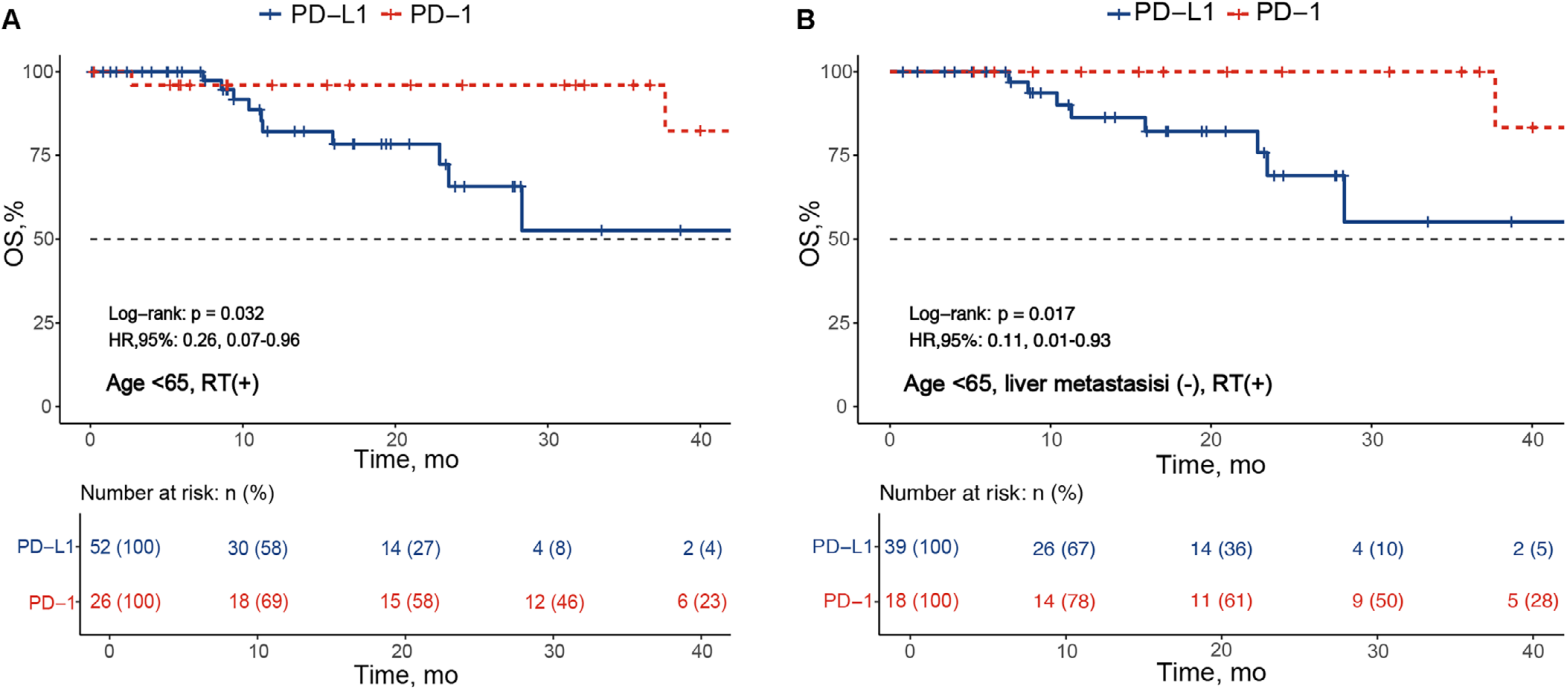
Kaplan–Meier subgroup analyses showing survival comparisons between anti‐PD‐1 and anti‐PD‐L1 therapy according to key prognostic factors including age, radiotherapy, and liver metastasis status. (A) Patients aged < 65 years with radiotherapy (RT+); (B) Patients aged < 65 years, no liver metastasis, with RT+.

We identified prognostic factors associated with OS and PFS and explored patient subgroups that might benefit preferentially from anti‐PD‐1 or anti‐PD‐L1 therapy. Univariate Cox regression was first performed for the clinical variables listed in Table 1. Variables significantly associated with OS included age, liver or bone metastasis, RT, neutrophil count, lymphocyte count, eosinophil percentage, serum albumin (ALB), lactate dehydrogenase (LDH), ECOG performance status of 2 or greater, and first‐line IT. These variables were subsequently included in a multivariable Cox regression model. As shown in Table 2, age (*p* = 0.017), liver metastasis (*p* = 0.002), RT (*p* = 0.009), ALB (*p* = 0.010), and LDH (*p *= 0.012) remained independently associated with OS. For PFS, univariate analysis showed associations with liver metastasis, RT, neutrophil count, monocyte count, eosinophil count, ALB, LDH, and ECOG = 1. In the multivariable model, liver metastasis (*p* = 0.009), RT (*p *= 0.007), and LDH (*p* = 0.042) remained statistically significant predictors of PFS (Table 3). Kaplan–Meier curves for significant prognostic factors are presented in Figure S7. Further stratified analyses showed that, in the anti‐PD‐1 group, patients younger than 65 years and those who received RT had longer OS than corresponding patients in the anti‐PD‐L1 group; this OS benefit was particularly evident in patients without liver metastasis (Figure 3).

**FIGURE 4:**
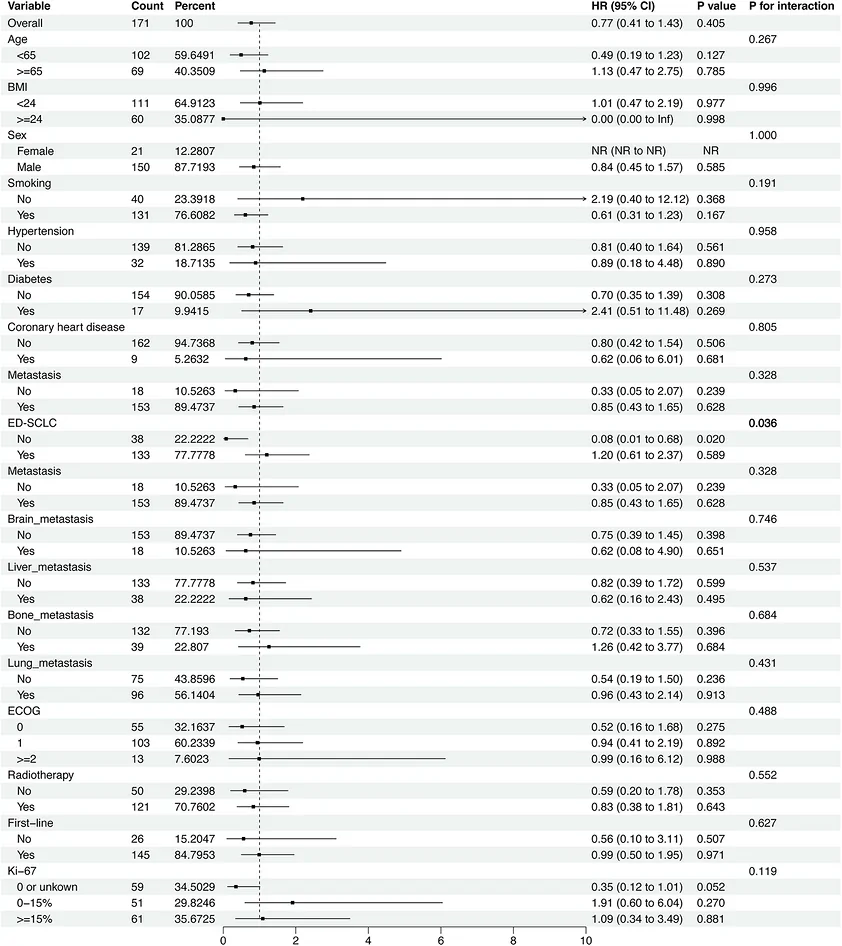
Forest plot of subgroup analyses for overall survival comparing anti‐PD‐1 therapy with anti‐PD‐L1 therapy across predefined clinical subgroups.

**FIGURE 5:**
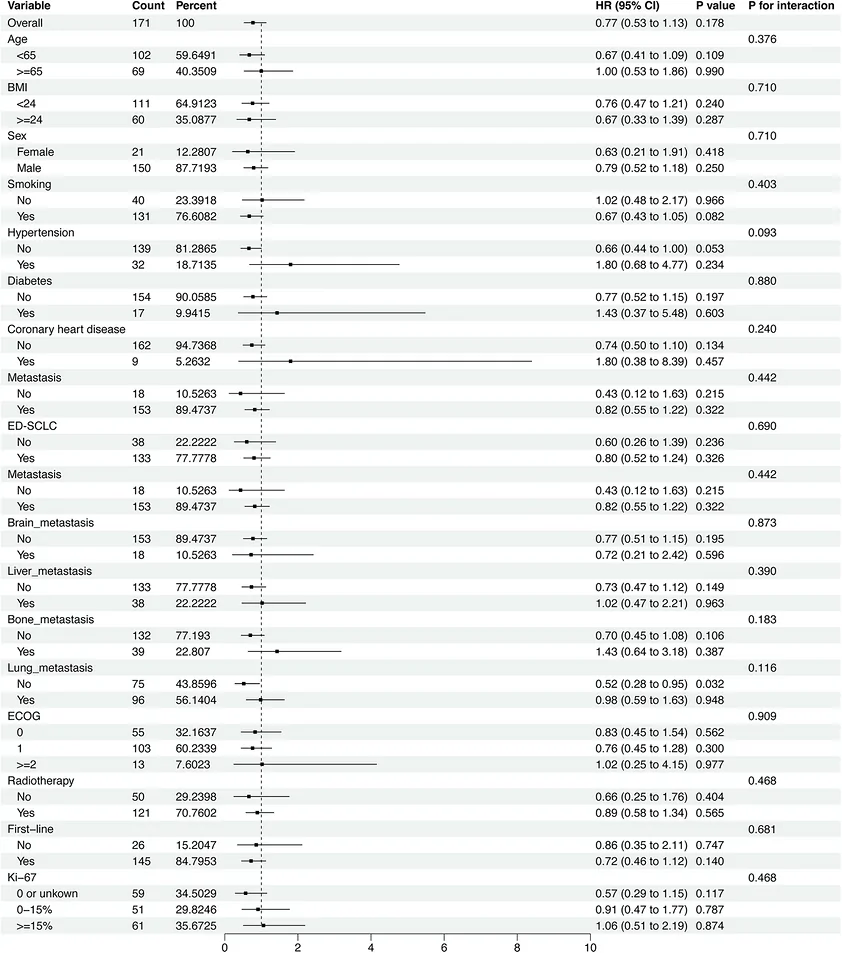
Forest plot of subgroup analyses for progression‐free survival comparing anti‐PD‐1 therapy with anti‐PD‐L1 therapy across predefined clinical subgroups.

**FIGURE 6:**
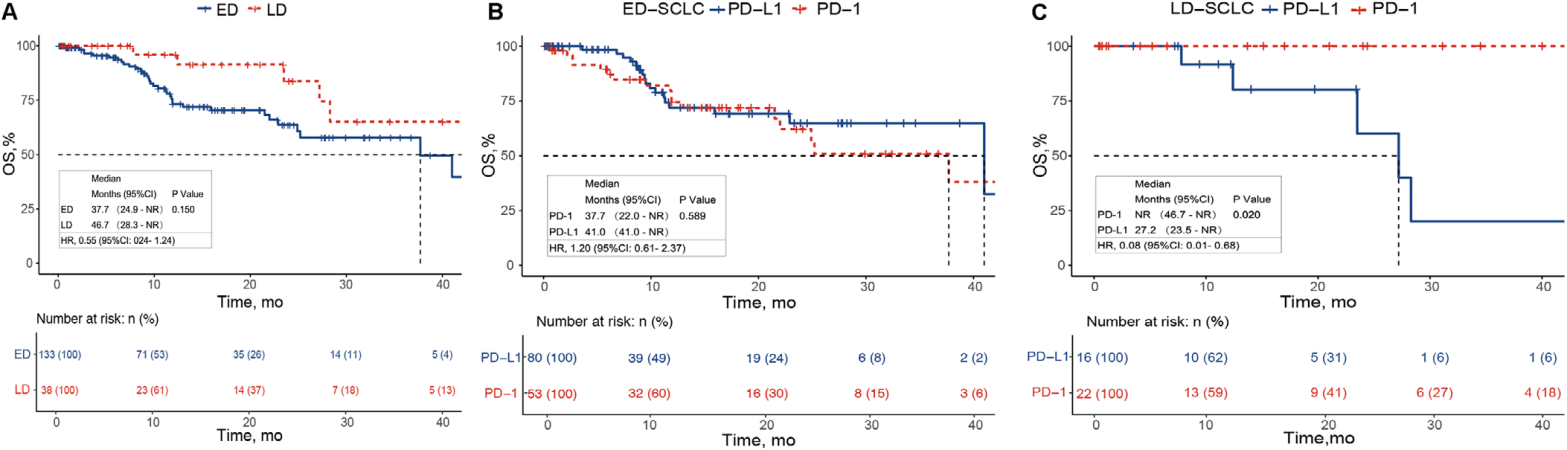
Kaplan–Meier curves of overall survival in patients with limited‐stage small cell lung cancer treated with anti‐PD‐1 or anti‐PD‐L1 therapy. (A) ED‑SCLC versus LD‑SCLC; (B) Anti‐PD‑L1 vs anti‐PD‑1 in ED‑SCLC; (C) Anti‐PD‑L1 vs anti‐PD‑1 in LD‑SCLC.

To evaluate the consistency of treatment effects across predefined subgroups, forest plots were constructed for OS and PFS (Figures 4 and 5). Anti‐PD‐1 therapy exerted a significantly protective effect on OS in patients with LD‐SCLC (HR, 0.08; 95% CI, 0.01–0.68; *p* = 0.020; *p* for interaction = 0.036), although no significant PFS advantage was observed (HR, 0.60; 95% CI, 0.26–1.39; *p* = 0.236; *p* for interaction = 0.690). In the LD‐SCLC subgroup, median OS was not reached in the anti‐PD‐1 group, whereas the median OS was 27.2 months in the anti‐PD‐L1 group (*p* = 0.020), indicating improved OS with anti‐PD‐1 therapy (Figure 6). Comparable OS and PFS outcomes were observed across the remaining subgroups. Taken together, these results suggest that LD‐SCLC may represent a population more likely to derive OS benefit from anti‐PD‐1 therapy.

### 2.4 Safety Analysis

Adverse events (AEs) were recorded in both treatment groups, and toxicity profiles are summarized in Table 4. All‐grade immune‐related adverse events (irAEs) and Grade 3 or higher non‐irAEs were extracted for comparison. Overall, 80 patients experienced AEs, including 50 patients (52.1%) in the anti‐PD‐L1 group and 30 patients (40.0%) in the anti‐PD‐1 group. Non‐irAEs were more common than irAEs. The overall incidence of any‐grade irAEs was similar between the two groups. Among patients who developed irAEs, dermatitis and pneumonia occurred relatively frequently. Grade 3 or higher irAEs occurred in 11 patients (11.4%) in the anti‐PD‐L1 group and 4 patients (5.3%) in the anti‐PD‐1 group. No statistically significant differences in irAEs or non‐irAEs were observed between groups. Overall, both anti‐PD‐1 and anti‐PD‐L1 therapies showed manageable safety profiles in this retrospective cohort.

**TABLE 2:** Univariate and multivariate analysis in OS.

	OS			
	Univariate		Multivariate	
	HR (95% CI)	*p* value	HR (95% CI)	*p* value
Age	1.11 (1.06–1.16)	< 0.001	**1.03 (1.01–1.07)**	**0.017**
ECOG ≥ 2	2.75 (1.07–7.08)	0.036	1.31 (0.55–3.11)	0.536
Liver metastasis	2.74 (1.38–5.43)	0.004	**2.23 (1.34–3.86)**	**0.002**
Bone metastasis	2.17 (1.14–4.15)	0.019	1.04 (0.62–1.74)	0.887
Radiotherapy	0.42 (0.22–0.82)	0.003	**0.42 (0.21–0.82)**	**0.009**
First‐line immunotherapy	2.59 (1.04–6.46)	0.041	0.80 (0.47–1.36)	0.405
Neutrophil count × 10^9^/L mean (SD)	1.05 (1.01–1.08)	0.003	1.02 (0.93–1.12)	0.397
Lymphocyte count × 10^9^/L mean (SD)	1.16 (1.03–1.29)	0.012	0.87 (0.60–1.26)	0.470
Eosinophil%	1.07 (1.01–1.13)	0.021	2.07 (0.48–8.91)	0.330
Serum albumin	1.01 (1.00–1.01)	0.019	**1.02 (1.00–1.04)**	**0.010**
Lactate dehydrogenase	2.75 (1.07–7.08)	0.003	**1.00 (1.00–1.00)**	**0.012**

*Note*: Bold text in multivariate: The results are statistically significant, with a *p* value of less than 0.05.

Abbreviations: ECOG: Eastern Cooperative Oncology Group, RT: Radiotherapy.

**TABLE 3:** Multivariate analysis in PFS.

	PFS			
	Univariate		Multivariate	
	HR (95% CI)	*p* value	HR (95% CI)	*p* value
ECOG = 1	0.62 (0.43–0.91)	0.013	0.70 (0.47–1.06)	0.096
Liver metastasis	2.39 (1.53–3.73)	< 0.001	**2.00 (1.18–3.40)**	**0.009**
RT	2.00 (1.19–3.35)	0.009	**2.25 (1.25–4.05)**	**0.007**
Neutrophil count × 10^9^/L mean (SD)	1.05 (1.01–1.08)	0.005	1.00 (0.91–1.11)	0.939
Monocyte count × 10^9^/L mean (SD)	1.22 (1.07–1.38)	0.004	1.32 (0.93–1.77)	0.062
Eosinophil count × 10^9^/L mean (SD)	2.79 (1.04–7.50)	0.021	1.89 (0.44–8.12)	0.392
Serum albumin	1.01 (1.00–1.01)	<0.001	0.98 (0.93–1.02)	0.318
Lactate dehydrogenase	1.00 (1.00–1.00)	<0.001	**1.00 (1.00–1.01)**	**0.042**

*Note*: Bold text in multivariate: The results are statistically significant, with a *p* value of less than 0.05.

Abbreviations: ECOG, Eastern Cooperative Oncology Group; RT, Radiotherapy.

**TABLE 4:** Adverse events.

	Any Grade			≥ 3 Grade		
	PD‐L1 (*n* = 50, 52.0%)	PD‐1 (*n* = 30, 40.0%)	*p* value	PD‐L1 (*n* = 11, 11.4%)	PD‐1 (*n* = 4, 5.0%)	*p* value
All	50 (52.1)	30 (40.0)	0.291	11 (11.5)	4 (5.3)	0.373
irAEs						
All	14 (28.0)	8 (26.7)	0.035	6 (54.5)	1 (25.0)	0.109
Dermatitis	2 (4.0)	3 (10.0)	0.562	1 (9.1)	0 (0)	0.823
Pneumonia	5 (10.0)	4 (13.3)	0.901	3 (27.3)	1 (25.0)	0.996
Cardiac dysfunction	2 (4.0)	0 (0)	0.540	0 (0)	0 (0)	0.045
Adrenal insufficiency	0 (0)	0 (0)	NA	0 (0)	0 (0)	0.045
Kidney insufficiency	2 (4.0)	1 (3.3)	0.989	1 (9.1)	0 (0)	0.823
Nervous system disorder	2 (4.0)	1 (3.3)	0.989	1 (9.1)	0 (0)	0.823
Thyroid dysfunction	0(0)	3 (10.0)	0.074	0 (0)	0 (0)	0.045
Steroids use due to irAEs	2 (4.0)	3 (10.0)	0.562	0 (0)	0 (0)	0.045
Endocrine disorder	0 (0)	0 (0)	NA	0 (0)	0 (0)	NA
Colitis	0 (0)	0 (0)	NA	0 (0)	0 (0)	0.045
Allergic events	1 (2.0)	2 (6.7)	0.568	0 (0)	0 (0)	0.045
Non‐irAEs						
All	44 (88.0)	27 (90.0)	0.999	9 (81.8)	4 (100.0)	0.802
Myelosuppression	28 (56.0)	14 (46.7)	0.721	8 (72.7)	4 (100.0)	0.506
Anemia	2 (4.0)	4 (13.3)	0.308	0 (0)	1 (25.0)	0.229
Febrile	3 (6.0)	7 (23.3)	0.076	0 (0)	0 (0)	0.045
Vomiting/nausea	10 (20.0)	2 (6.7)	0.271	1 (9.1)	0 (0)	0.823
Diarrhea	0 (0)	0 (0)	NA	0 (0)	0 (0)	0.045
Cough	2 (4.0)	5 (16.7)	0.152	1 (9.1)	0 (0)	0.823
Infectious events	4 (8.0)	5 (16.7)	0.494	2 (18.2)	0 (0)	0.657
Liver dysfunction	2 (4.0)	1 (3.3)	0.989	0 (0)	1(25.0)	0.229
Digestive dysfunction	4 (8.0)	3 (10.0)	0.954	0 (0)	0 (0)	0.045
Others	6 (16.0)	1 (3.3)	0.222	3 (27.3)	0 (0)	0.506

*Note*: NA: No patients had this adverse event, no *p* value could be calculated.

Abbreviations: irAE, immune‐related adverse event; NA, not available.

## 3 Discussion

SCLC is an aggressive smoking‐related malignancy that accounts for approximately one‐seventh of all lung cancer cases. It is characterized by high initial sensitivity to CT and RT; however, early recurrence and distant metastasis are common. Consequently, SCLC poses a substantial therapeutic challenge and is associated with an extremely poor prognosis [12, 13]. With the advent of IT, ICIs have revolutionized the treatment landscape of SCLC and offered promising new therapeutic strategies [14].

This retrospective two‐center study was designed to compare the efficacy and safety of anti‐PD‐1 and anti‐PD‐L1 therapies in patients with SCLC. In our cohort, no meaningful differences in OS or PFS were observed between the two treatment groups, although the incidence of irAEs appeared numerically higher in the anti‐PD‐L1 group. The median OS and PFS in the anti‐PD‐1 group were 46.7 months and 11.4 months, respectively, which were numerically longer than those in the anti‐PD‐L1 group, but the differences did not reach statistical significance. Combination treatment with RT was associated with prolonged OS compared with treatment without RT.

Subgroup analysis further demonstrated that patients younger than 65 years, those receiving IT combined with RT, and those with LD‐SCLC derived greater survival benefit from anti‐PD‐1 treatment than from anti‐PD‐L1 treatment. Although the anti‐PD‐L1 group exhibited a higher rate of Grade 3 or higher AEs, this difference was not statistically significant. Notably, previous studies have yielded inconsistent results regarding the comparative therapeutic efficacy of anti‐PD‐1 and anti‐PD‐L1 agents, which may be partly attributable to the relatively small sample size in each study cohort [15, 16, 17].

Currently, most IT studies in SCLC have focused on the ED‐SCLC setting. A meta‐analysis by Chen et al. indicated that, in first‐line treatment for ED‐SCLC, both anti‐PD‐1 and anti‐PD‐L1 therapies improve survival outcomes compared with CT alone. Indirect comparisons of OS and PFS among atezolizumab, durvalumab, pembrolizumab, and nivolumab showed no statistically significant differences, which is consistent with our findings [18]. In contrast, therapeutic progress in LD‐SCLC has remained stagnant for decades, and the role of anti‐PD‐1 therapy in patients receiving concurrent chemoradiotherapy remains incompletely understood [19]. Our study suggested that patients with LD‐SCLC exhibited improved OS in the anti‐PD‐1 group compared with the anti‐PD‐L1 group. A Phase 1/2 trial by Welsh et al. suggested that concurrent pembrolizumab plus chemoradiotherapy was well tolerated and associated with favorable OS and PFS in patients with LD‐SCLC, supporting its safety and efficacy [20, 21]. Furthermore, a Phase 3 randomized clinical trial indicated that durvalumab combined with chemoradiotherapy improved OS in patients with LD‐SCLC [22].

In line with these findings, our results confirmed that combination with RT was associated with prolonged OS. Notably, among younger patients without liver metastasis, the survival advantage of anti‐PD‐1 was significantly greater than that of anti‐PD‐L1. Several mechanisms may underlie this difference. PD‐1 antibodies block PD‐1 on tumor‐infiltrating T lymphocytes, thereby interrupting interactions with both PD‐L1 and PD‐L2 and leading to more complete inhibition of immune escape. By contrast, anti‐PD‐L1 antibodies block only the PD‐1/PD‐L1 axis, theoretically allowing residual immune evasion via PD‐L2 [23]. In addition, the liver is an immune‐tolerant organ characterized by functionally impaired T cells and a strongly immunosuppressive microenvironment, which may reduce the efficacy of IT [24]. Variations in treatment regimens and clinical management may also have contributed to the observed differences. Therefore, the survival differences between anti‐PD‐1 and anti‐PD‐L1 in specific subgroups should be interpreted with caution, and further validation is required. Our study systematically explored differences in therapeutic efficacy between anti‐PD‐1 and anti‐PD‐L1 in SCLC, with a focus on survival stratification between ED‐SCLC and LD‐SCLC, aiming to identify populations that may achieve the greatest benefit. This study supplements the current literature by providing direct comparative evidence on anti‐PD‐1 versus anti‐PD‐L1 agents while simultaneously assessing their safety profiles.

Several limitations should be noted in the present study. First, the relatively modest sample size may have reduced the statistical power, particularly in subgroup analyses. Second, as a retrospective study, selection bias and confounding factors were unavoidable. Thus, large‐scale prospective and randomized trials are needed to confirm these findings. Third, due to limited available data, we were unable to analyze molecular markers, including PD‐L1 expression, the Ki‐67 index, and genetic alterations, which should be incorporated into future studies.

## 4 Conclusions

In summary, both anti‐PD‐1 and anti‐PD‐L1 agents improve the prognosis of SCLC. There was no survival difference between the two treatments in the entire cohort. However, prognosis was significantly better in patients younger than 65 years who received RT and in patients with LD‐SCLC in the anti‐PD‐1 group compared with the anti‐PD‐L1 group. These findings warrant further investigation to identify patient populations that may benefit from anti‐PD‐1 treatment.

## 5 Methods

### 5.1 Patient Selection

This study enrolled patients with SCLC who received anti‐PD‐1 or anti‐PD‐L1 therapy at West China Hospital and West China Lung Cancer Center, Sichuan University, between January 2016 and June 2023. The study was approved by the Biomedical Research Ethics Committee of West China Hospital (No. 20241410). Written informed consent was waived due to the retrospective design.

The inclusion criteria were as follows: (1) histopathologically confirmed primary SCLC, with tumor stage clearly defined according to the VALG staging system [25]; (2) receipt of anti‐PD‐1 or anti‐PD‐L1 therapy, with the specific drug name and regimen clearly recorded; (3) complete diagnosis and treatment data; (4) absence of other primary tumors; (5) age 18 years or older; and (6) complete baseline information. The exclusion criteria were as follows: (1) diagnosis of another malignant tumor prior to lung cancer, (2) diagnosis limited to lung metastases without clear identification of the primary tumor, (3) active autoimmune disease, (4) previous organ transplantation or ongoing immunosuppressive therapy, (5) uncontrolled active infection, (6) severe lack of baseline data affecting eligibility confirmation or efficacy comparison, and (7) inability to evaluate clinical status. The primary outcomes were OS and PFS. OS was defined as the period from SCLC diagnosis to death or last follow‐up, whereas PFS was defined as the time from initial SCLC diagnosis to tumor progression, death, or last follow‐up. The follow‐up cutoff date was June 30, 2024, and survival status was documented through the electronic medical record system or telephone follow‐up.

### 5.2 Data Collection

For all eligible patients included in the final analysis, laboratory test results at the time of SCLC diagnosis were extracted from the hospitalization record system. The collected data included baseline characteristics (sex, age, BMI, smoking history, underlying diseases, and ECOG performance status), tumor characteristics (Ki‐67 expression, PD‐L1 expression, disease stage, metastatic sites, and treatment plans), and blood test results. Treatment regimens were divided into four types: IT, IT plus CT, IT plus RT, and IT plus CT plus RT. The definitions were as follows: IT, IT alone; IT plus CT, receipt of IT and CT administered concurrently or sequentially without RT; IT plus RT, receipt of IT and RT without CT; and IT plus CT plus RT, receipt of IT, CT, and RT administered concurrently or sequentially.

### 5.3 Statistical Analysis

Continuous variables were reported as mean with standard deviation or median with interquartile range where indicated. Categorical variables were summarized using counts and percentages. Intergroup comparisons used the Wilcoxon rank‐sum test or chi‐square test [26]. The Kaplan–Meier method was used to estimate survival time, and between‐curve differences were assessed with the log‐rank test [27]. Cox proportional hazards regression analyses were conducted, and results were presented as HRs with corresponding 95% CIs [28]. Furthermore, stratified subgroup analyses were carried out to examine the consistency of treatment effects across different clinicopathological characteristics and to identify specific patient populations that might gain the greatest benefit from immunotherapy [29]. Two‐sided tests were applied for all analyses, with *p* < 0.05 considered statistically significant. All computations were performed using R software, version 4.2.2.

## Author Contributions

**Ruiyuan Yang**: conceptualization, data curation, methodology, software, writing – original draft, writing – review and editing. **Jiadi Gan**: conceptualization, data curation, methodology, visualization, writing – original draft, writing – review and editing. **Lan Yang**: conceptualization, data curation, methodology, visualization, writing – original draft, writing – review and editing. **Bojiang Chen**: conceptualization, methodology, writing – review and editing. **Weimin Li**: conceptualization, funding acquisition, methodology, project administration, writing – review and editing. All authors have read and approved the final manuscript

## Funding

This work was supported by the Noncommunicable Chronic Diseases‐National Science and Technology Major Project grant from the National Natural Science Foundation of China (grant no.: 2023ZD0506101/2023ZD0506100 to W. Li), the Fundamental Research Funds for the Central Universities (grant no.: SCU2024D017/SCU2025D017 to W. Li), the Science Fund for Distinguished Young Scholars grant from the Sichuan Natural Science Foundation of Science (grant no.: 2024NSFJQ0051 to B. Chen), and the Natural Science Foundation of Sichuan Province (grant no.: 2024NSFSC1909 to R. Yang).

## Ethics Statement

This retrospective study was approved by the Ethics Committee on Biomedical Research of West China Hospital (Number: 20241410). The requirement for informed consent was waived by the Ethics Committee due to the retrospective nature of this study.

## Conflicts of Interest

The authors declare no conflicts of interest.

## Supporting information




**Supporting File 1**: mco270872‐sup‐0001‐SuppMat.docx

## Data Availability

The raw data supporting the conclusions of this article will be made available by the authors on request.
