# Supplementary material for: Immunotherapy With Anti‐PD‐1 Versus Anti‐PD‐L1 in SCLC: A Comprehensive Retrospective Analysis
Source: MedComm (2020). 2026 Sep 11;7(9):e70872. doi: 10.1002/mco2.70872 (PMC13570190; doi:10.1002/mco2.70872)
Supplement: Supplementary file 1 [file MCO2-7-e70872-s001.docx]

**Immunotherapy with Anti-PD-1 vs Anti-PD-L1 in SCLC: A Comprehensive Retrospective Analysis**

Ruiyuan Yang, MD ^1,2,#^, Jiadi Gan, MD ^1,2,#^, Lan Yang, MD ^1.3†^, Bojiang Chen, MD, PhD ^1,4*^, Weimin Li, MD, PhD ^1,2,3,4,5,6*^

^1^ Department of Pulmonary and Critical Care Medicine, West China Hospital, Sichuan University, Chengdu 610041, Sichuan, China

^2^ State Key Laboratory of Respiratory Health and Multimorbidity, West China Hospital, Chengdu 610041, Sichuan, China

^3^ Institute of Respiratory Health, Frontiers Science Center for Disease-Related Molecular Network, West China Hospital, Sichuan University, Chengdu 610041, Sichuan, China

^4^ Precision Medicine Center, Precision Medicine Key Laboratory of Sichuan Province, West China Hospital, Sichuan University, Chengdu 610041, Sichuan, China

^5^ The Research Units of West China, Chinese Academy of Medical Sciences, West China Hospital, Chengdu 610041, Sichuan, China

^6^ Institute of Respiratory Health and Multimorbidity, West China Hospital, Sichuan University, Chengdu 610041, Sichuan, China

^#^ These authors contributed equally to this work.

***Correspondence:**

Weimin Li, Email: [weimin003@163.com](mailto:weimin003@163.com)

Bojiang Chen,Email: cjhcbj@outlook.com

Tel: +86 28 85423001; Fax: +86 28 85423001;

Address: Department of Respiratory and Critical Care Medicine, West China Hospital, Sichuan University, Chengdu, Sichuan, China

**Figure S1.** Kaplan-Meier Survival Curves of OS for different groups based on the basic characteristics. **A.** In aged ≥ 65 years subgroup. **B.** In aged < 65 years subgroup. **C.** In male subgroup. **D.** In female subgroup. **E.** In BMI ≥ 24 years subgroup. **F.** In BMI < 24 years subgroup. OS: overall survival, HR: Hazard ratio, CI: confidence interval, NR: not reported, BMI: body mass index.

**Figure S2.** Kaplan-Meier Survival Curves of OS for different groups based on the basic characteristics, including age, sex and BMI. **A.**PFS in aged ≥ 65 years subgroup **B.** In aged < 65 years subgroup. **C.** In male subgroup. **D.** In female subgroup. **E.** In BMI ≥ 24 kg/m^2^ subgroup. **F.** In BMI < 24 kg/m^2^ subgroup. PFS: progression free survival, HR: Hazard ratio, CI: confidence interval, NR: not reported, BMI: body mass index.

**Figure S3.** Kaplan-Meier Survival Curves regarding smoking status in anti-PD-1 and anti-PD-L1 group. **A.** The OS in smoking population. **B.** The PFS in smoking population. **C.** The OS in non-smoking population. **D.** The PFS in non-smoking population. PD-1: Programmed Death-1, PD-L1: Programmed Death-1 legend, OS: overall survival, PFS: progression free survival, HR: hazard ratio, CI: confidence interval, NR: not reported.

**Figure S4.** Kaplan-Meier Survival Curves of OS for different groups based on the underlying diseases. **A.** In diabetes subgroup. **B.** In non-diabetes subgroup. **C.** In hypertension subgroup. **D.** In non- hypertension subgroup. **E.** In CHD subgroup. **F.** In non-CHD subgroup. OS: overall survival, HR: Hazard ratio, CI: confidence interval, NR: not reported, CHD: coronary heart disease.

**Figure S5.** Kaplan-Meier Survival Curves of PFS for different groups based on the underlying diseases. **A.** In diabetes subgroup. **B.** In non-diabetes subgroup. **C.** In hypertension subgroup. **D.** In non- hypertension subgroup. **E.** In CHD subgroup. **F.** In non-CHD subgroup. PFS: progression free survival, HR: Hazard ratio, CI: confidence interval, NR: not reported, CHD: coronary heart disease

**Figure S6.** Kaplan-Meier Survival Curves between IT+CT and IT+CT+RT treatment, and the subgroups for comparing anti-PD-1 and anti-PD-L1. **A.** The OS in IT+CT group and IT+CT+RT group. **B.** The PFS in IT+CT group and IT+CT+RT group. **C.** The OS comparison for anti-PD-1 and anti-PD-L1 in IT+CT group. **D.** The PFS comparison for anti-PD-1 and anti-PD-L1 in IT+CT group. **E.** The OS comparison for anti-PD-1 and anti-PD-L1 in IT+CT+RT group. **F.** The PFS comparison for anti-PD-1 and anti-PD-L1 in IT+CT+RT group. PD-1: Programmed Death-1, PD-L1: Programmed Death-1 legend, OS: overall survival, PFS: progression free survival, HR: hazard ratio, CI: confidence interval, IT: immunotherapy, CT: chemotherapy, RT: radiotherapy, NR: not reported.

**Figure S7.** Kaplan-Meier Survival Curves of the risk factors derived from the multivariate Cox regression analysis of OS and PFS. **A.** OS in age subgroup. **B.** PFS in age subgroup. **C.** OS in RT subgroup. **D.** PFS in RT subgroup. **E.** OS in liver metastasis subgroup **F.** PFS in liver metastasis subgroup **G.** OS in ALB<40g/L subgroup. **H.** PFS in ALB<40g/L subgroup. **I.** OS in LDH>220 IU/L subgroup. **J.** PFS in LDH>220 IU/L subgroup. OS: overall survival, PFS: progression free survival, HR: hazard ratio, CI: confidence interval, RT: radiotherapy, NR: not reported, ALB: serum albumin, LDH: lactate dehydrogenase.
